# Infrarenal Aorta Thrombosis Associated with H1N1 Influenza A Virus Infection

**DOI:** 10.1155/2016/9567495

**Published:** 2016-10-31

**Authors:** Can Hüzmeli, Mustafa Saglam, Ali Arıkan, Barıs Doner, Gulay Akıncı, Ferhan Candan

**Affiliations:** ^1^Department of Nephrology, Necip Fazıl City Hospital, Kahramanmaras, Turkey; ^2^Department of Cardiovascular Surgery, Necip Fazıl City Hospital, Kahramanmaras, Turkey; ^3^Department of Anesthesia and Reanimation, Necip Fazıl City Hospital, Kahramanmaras, Turkey; ^4^Department of Nephrology, Cumhuriyet University, Sivas, Turkey

## Abstract

Influenza viruses are members of the Orthomyxoviridae family, of which influenza A, B, and C viruses constitute three separate genera. Arterial thrombosis associated with H1N1 influenza A virus infection has rarely been reported. A Turkish man aged 28 years was admitted to our emergency department with dyspnea, bilateral lower extremity insensitivity, and cold. He reported symptoms of fever, myalgia, and cough, which he had had for fifteen days before being admitted to our hospital. The patient was tested for pandemic influenza A (H1N1) virus using polymerase chain reaction (PCR) tests, which were positive. Abdominal computerized tomography with contrast revealed a large occlusive thrombus within the infrarenal aorta.

## 1. Introduction

The difference in thrombus formation in arterial and venous systems indicates that there are different etiologies in these two different systems. It is known that disorders in the stasis and coagulation systems lead to thrombosis development in the venous system, and endothelium damage and functional disorders in thrombocytes play an instrumental role in the arterial system [1].

During infection, possible thrombosis mechanisms include increased thrombocyte activation and changes in procoagulant and anticoagulant factor balance including stasis and vascular endothelial dysfunction or activation secondary to immobilization. Arterial thrombosis generation can differ from pathogenic venous thrombosis and usually occurs as a result of endovascular damage or endothelial damage [2].

We describe a patient with heart failure who developed an infrarenal aortic thrombosis and viral pneumonia during an H1N1 infection.

## 2. Case Presentation

A man aged 28 years was admitted to our clinic with symptoms of shortness of breath, cough, numbness, pain, and coldness in the lower extremity. His medical history showed that he had heart failure and he had a pacemaker. Approximately 15 days before presenting to our clinic, he had symptoms of fatigue, cough, sore throat, and fever. In the physical examination, he was found to have 130/70 mm Hg blood pressure, 20/min respiratory rate, 37°C body temperature, 108/min pulse rate, and bilateral, crepitant rales in his lungs, and bilateral lower extremity pulses were not detected.

**Figure 1:**
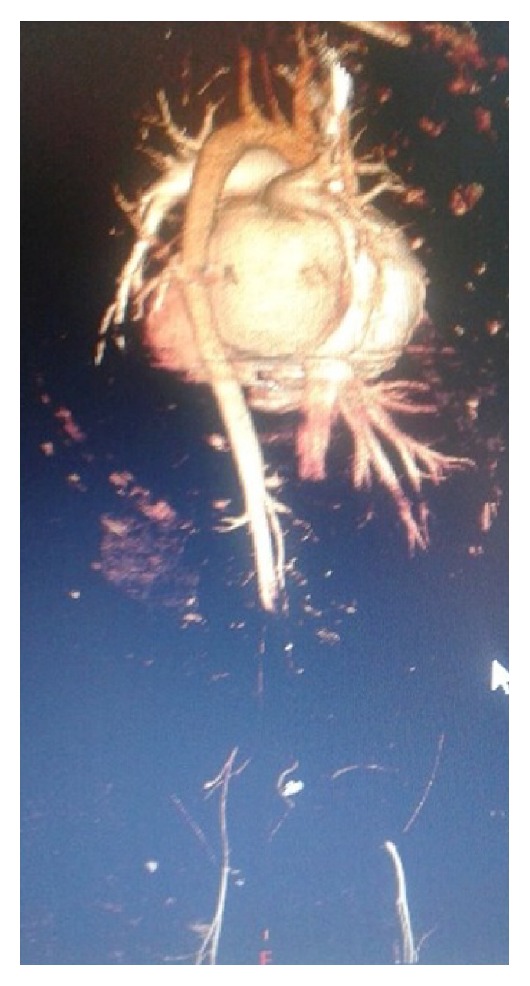
Abdominal computed tomography with contrast demonstrated a large occlusive thrombus within the infrarenal aorta, thrombus in bilateral iliac artery, and focal hypodense areas in the left external right kidney parenchyma.

The laboratory examination revealed the following values: glucose 146 mg/dL (100–125 mg/dL), blood urea nitrogen (BUN) 18 mg/dL (9–23 mg/dL), serum creatinine (CRE) 1.4 mg/dL (0.6–1.2 mg/dL), total protein 6.4 gr/dL (5.7–8.2 gr/dL), albumin 3.8 gr/dL (3.2–4.8 gr/dL), amylase 159 IU/L (28–100 IU/L), alkaline phosphatase 94 IU/L (35–105 IU/L), alanine aminotransferase (ALT) 24 IU/L (0–32 IU/L), aspartate aminotransferase (AST) 36 IU/L (0–33 IU/L), lactate dehydrogenase (LDH) 4121 IU/L (135–214 IU/L), creatinine kinase (CK) 908 IU/L (26–192 IU/L), gamma glutamyl transferase 62 IU/L, sodium 132 mEq/L (135–145 mEq/L), potassium 4.2 mEq/L (3.5–5.5 mEq/L), erythrocyte sedimentation rate 18 mm/hr, CRP 154 mg/L (0–8 mg/L), white cell count 15 000 mm^3^/*μ*L, neutrophil 89%, lymphocyte 3%, hemoglobin (Hb) 13 gr/dL (13–17 gr/dL), Htc 41% (36–48%), and platelet count 280 000/mm^3^ (150 000–400 000/mm^3^). Arterial blood gas was metabolic acidosis (pH 7.29, PO_2_ 76 mm Hg, PCO_2_ 34 mm Hg, HCO_3_ 18 mEq/L). Abdominal computerized tomography (CT) angiography was performed to test for thromboses, which revealed a thrombosis in the infrarenal aorta (Figure 1), and thorax CT angiography revealed pleural effusions of 2.8 cm in the right hemithorax and 1.6 cm in the left hemithorax, prevalent ground-glass areas in the parenchyma of both lungs, and reticulonodular infiltrates showing nodule formation, which were dominant in the upper lobe of the left lung but prevalent in both lungs.

Patient was presented with instantaneous severe lower limb pain, coolness, paleness, and absence of pulses and CT angiography confirmed infrarenal aorta thrombosis. Therefore, we decided to perform emergency surgery. Under local anesthesia bilateral common femoral artery exposed and femoral embolectomies were performed after the administration of heparin, and a large amount of fresh thrombus material was retrieved. Immediately after femoral circulation was restored. After the operation the patient was transferred to intensive care unit.

In the postop laboratory evaluations the following results were obtained: glucose 139 mg/mL, BUN 41 mg/dL, CRE 4 mg/dL, ALT 103 IU/L, AST 641 IU/L, CK 11915 IU/L, LDH 2625 IU/L (135–225 IU/L), sodium 129 mEq/L, potassium 6.0 mEq/L, INR 2.2, leucocyte count 17000/mm^3^, Hb 10 gr/dL, Htc 30, and Plt 187 000 mm^3^ (lymphopenia presence 3%). Urinalysis was not performed because the patient was anuric. Enoxaparin treatment was initiated due to thrombosis. Empirical moxifloxacin treatment was given owing to the prediagnosis of pneumonia. No growth was reported in the blood culture. The patient was diagnosed as having anuric acute kidney injury (AKI), Kidney Disease: Improving Global Outcomes (KDIGO) stage 3 and taken into hemodialysis after catheterization. Autoantibody analysis was requested because of the AKI etiology and the results (P-ANCA, C-ANCA, anti-Ds DNA, and ANA) were negative with negative components and negative rheumatoid factor. In the analyses requested to evaluate thrombosis, anti-thrombin III and protein C values were normal, protein S 50% (55–160) was slightly low, and anticardiolipin and antiphospholipid antibody results were negative. Serum* Legionella pneumophila* was negative in the patient who had hyponatremia (sodium 129 mmol/L) and pneumonia.

PCR analysis conducted on nose and throat swap samples was positive for H1N1. Oseltamivir was added to the patient's treatment. Four units of erythrocyte suspension were given upon a decrease in the patient's Hb value. Fever was detected in the follow-up examination conducted two weeks after the initiation of treatment and cultures were taken from the patient. Piperacillin-tazobactam was added to the treatment. On the 17th day of hospitalization, the patient had sudden shortness of breath and the following clinical values: blood pressure 80/30 mm Hg, body temperature 37°C, respiratory rate 30/min, and pulse 108/min, and rales were present in his lungs. Growth of* Acinetobacter baumannii* was observed in a blood culture. Arterial blood gas was respiratory and metabolic acidosis (pH 7.11, PCO_2_ 65 mm Hg, PO_2_ 45 mm Hg, and HCO_3_ 15 mEq/L). The patient was evaluated to have sepsis and was intubated and connected to mechanical ventilation, and inotrope treatment was initiated. Despite the inotrope treatment, the patient was hypotensive and died on the 17th day of treatment.

## 3. Discussion

Clinically, H1N1 infections comprise symptoms of fever, cough, shortness of breath, myalgia, and headache. In some individuals, the infection may be mild and constrained. However, in others, the infection may be severe. In severe cases, respiratory failure, alveolar hemorrhage, rhabdomyolysis, and AKI damage can occur and may result in death. The most prevalent laboratory findings observed are high ALT and AST values, anemia, leucopenia, leukocytosis, thrombocytopenia, thrombocytosis, and high total bilirubin values. Furthermore, severe cases show mild and moderately high LDH and CK values [3, 4]. Our patient had the typical medical history of having H1N1 viral infection for 15 days. In addition, laboratory findings supported H1N1 viral infection (leukocytosis, lymphocytopenia, and high LDH and CK values).

It was thought that the patient may have viral pneumonia and/or alveolar hemorrhage; however, a differential diagnosis was not possible because bronchoscopy could not be conducted. In high resolution CT, H1N1 may include ground-glass opacities, consolidated areas, or both. These findings usually show bilateral and peripheral, subpleural, peribronchovascular, lobular, or random distribution. Pulmonary emboli lobar, segmental and subsegmental pulmonary arteries, and unilateral or bilateral pleural effusion are defined in these patients. Alveolar hemorrhage is also observed, related to the viral infection [5, 6].

Seasonal influenza virus infections are related to vascular events and thrombosis. Although vascular events were shown to increase in viral influenza infections in one study, another study reported an important connection between seasonal influenza infections and venous thromboembolism [7, 8].

In a study in which Bunce et al. observed 119 patients with H1N1, thrombotic vascular events were detected in 7 patients. Two of these 7 patients had myocardial infarction (one died), 3 had venous thrombosis, 1 had pulmonary emboli, and 1 had an infrarenal aorta thrombosis. In one case, the patient was in intensive care unit due to respiratory failure receiving mechanical ventilation, abdominal CT was conducted after a pulse was not found bilaterally in the lower extremities, and intrarenal aorta thrombosis was detected. In the treatment of this patient, embolectomy, bilateral aortoiliac stents, and leg amputation were performed to the left upper knee [9]. In a study conducted by Avnon et al., 5 out of 20 (25%) patients had thrombotic events [10].

In a study by Patel et al., a total of 1071 patients with H1N1 cases were included. AKI was observed in 208 of these patients. The causes of AKI in these patients were defined as sepsis, prerenal causes, medicinal, and secondary bacterial infections. Renal replacement treatment was initiated in 44 patients and 96 (48%) patients died [11]. Another study detected 25 patients with AKI out of 47 patients with H1N1, among whom 9 died; no AKI or deaths were reported in the remaining 22 patients [12]. The frequent occurrence of rhabdomyolysis in adult patients with H1N1 viral infections is widely recognized. In a study conducted in Mexico on 18 severe cases, high creatinine kinase values were observed in more than 60% of the patients [13]. In our case, thrombosis, contrast nephropathy, and rhabdomyolysis were suspected as AKI; however, sepsis was later added to these findings.

Patients with H1N1 usually recover by themselves without complications. H1N1 treatment is given to those with complications who have high mortality. The World Health Organization (WHO) recommends use of oseltamivir in the treatment of severe, complicated or progressive, or possibly pandemic H1N1 influenza cases, because of oseltamivir's ease of use and its systemic effect. The WHO also recommends zanamivir only in situations where oseltamivir is not available or the virus is resistant to oseltamivir [14].

As a result, arterial thrombosis is rarely seen in H1N1 infections. Clinically, arterial thrombosis is important regarding morbidity and mortality. Affected individuals show that possible hypercoagulation, endothelium activation, or dysfunction is related to H1N1.
